# Real-time search-assisted multiplexed quantitative proteomics reveals system-wide cryptic translation initiation in human cancer cells

**DOI:** 10.1186/s13059-026-04120-z

**Published:** 2026-05-28

**Authors:** Hiroko Kozuka-Hata, Tomoko Hiroki, Aya Kitamura, Naoaki Miyamura, Tetsu Akiyama, Jun-ichiro Inoue, Kouhei Tsumoto, Masaaki Oyama

**Affiliations:** 1 Medical Proteomics Laboratory, The Institute of Medical Science, The University of Tokyo, 4-6-1, Shirokanedai, Minato-Ku, Tokyo, 108-8639 Japan; 2 Laboratory of Molecular and Genetic Information, Institute for Quantitative Biosciences, The University of Tokyo, 1-1-1, Yayoi, Bunkyo-Ku, Tokyo, 113-0032 Japan; 3 The University of Tokyo Pandemic Preparedness, Infection and Advanced Research Center (UTOPIA), 4-6-1, Shirokanedai, Minato-Ku, Tokyo, 108-8639 Japan; 4 Department of Bioengineering, Graduate School of Engineering, The University of Tokyo, 7-3-1, Hongo, Bunkyo-Ku, Tokyo, 113-8656 Japan

## Abstract

**Background:**

It is generally considered that eukaryotic translation initiation prominently occurs from the first AUG codon by ribosomal scanning from the 5-cap end of each mRNA. In order to identify cryptic internal translation initiation sites defined by alternative AUG codons on a proteome-wide scale, we generate a customized amino acid sequence database which contain differential AUG-guided tryptic peptide fragments computationally predicted from well-curated Swiss-Prot human protein reference sequences and applied it for high-resolution mass spectrometry-based proteomic analysis.

**Results:**

The ultra-deep proteomic detection based on the real-time search platform on Orbitrap Eclipse Tribrid mass spectrometry system leads to identification of not only more than 26,000 unique peptides from already annotated human protein coding sequences but also 794 novel peptide fragments defined by alternative downstream translation initiation in human cancer cells. Very notably, Tandem Mass Tag-based multiplex quantitative analysis of patient-derived glioblastoma initiating cells uncovers epidermal growth factor-dependent translational regulation on a wide range of differential AUG-guided non-canonical proteoforms as well as cancer-related transcription factors and cell cycle/cell division regulators in a cell-type specific manner.

**Conclusions:**

Our study provides the first proteome-wide evidence of downstream AUG-guided cryptic translation initiation dynamics in human cancer cells.

**Supplementary Information:**

The online version contains supplementary material available at 10.1186/s13059-026-04120-z.

## Background

According to the conventional translation initiation model, a 40S ribosomal subunit is first recruited to the cap structure of mRNA and linearly scans the 5′-untranslated regions (UTRs) for the initiator codon. It is generally considered that, when it encounters the first AUG codon, it pauses until a large 60S ribosomal subunit joins and the complete ribosomal complex starts translation [1]. In parallel with human genome sequencing projects [2, 3], large-scale accumulation of human transcriptome sequences has also been performed and the longest open reading frame (ORF) on each transcript is manually curated and defined as the protein-coding sequence (CDS) in public databases such as Swiss-Prot (The UniProt Knowledgebase) and RefSeq (The National Center for Biotechnology Information) [4, 5]. In addition to the curated protein sequence information, high-resolution mass spectrometry-based proteomics has also unveiled the existence of translated products from numerous upstream ORFs which were previously presumed as ‘non-coding’ nucleotide regions based on the conventional rule of translation initiation [6, 7]. Although a series of previous studies revealed some biological mechanisms by which the ribosomal complex may initiate translation from alternative downstream codons, such as leaky scanning and IRES (internal ribosome entry site)-dependent translation [8, 9], there have been no reports to describe a comprehensive landscape of alternative downstream AUG-guided translation initiation dynamics activated in human cells. In order to perform mass spectrometry-based annotation of cryptic translation initiation sites defined by alternative downstream AUG codons on a proteome-wide scale, we newly generated a customized amino acid sequence database of differential AUG-guided tryptic peptide fragments based on well-curated Swiss-Prot human protein sequences and applied them for this specialized database-dependent novel peptide identification.

Our previous phosphoproteomic analysis of patient-derived glioblastoma initiating GB2 cells revealed EGF-induced drastic upregulation of RPS6 phosphorylation in comparison with well-studied human cervical cancer HeLa cells [10, 11]. In order to analyze the system-wide effect of EGF-dependent proteome regulation in these cancer cells, we performed a Tandem Mass Tag (TMT)-based multiplex quantitative analysis of the whole cellular proteome dynamics in the presence/absence of mTOR inhibitor Torin 1, which is well-known to contribute to regulate translational initiation through functional suppression of mTORC complex [12]. The ultra-deep mass spectrometric analysis based on the advanced real-time search (RTS) platform on Orbitrap Eclipse Tribrid mass spectrometry system led to identification of not only more than 26,000 peptides from already annotated Swiss-Prot human protein coding sequences but also approximately 800 non-canonical peptide fragments defined by alternative downstream AUG-guided translation initiation [13–15]. Very notably, TMT-based large-scale multiplexed quantification of GB2 cellular proteome dynamics in response to prolonged EGF treatment unveiled mTOR signaling-dependent prominent translational upregulation of non-canonical alternative proteoforms as well as glioblastoma-related transcription regulators (EWSR1, GABPB1) [16, 17] and cell cycle/cell division regulators (PCNA, Condensin complex subunits, MCM complex proteins) [18–22] in comparison with human HeLa cells. Our study provided the first system-wide landscape of differential downstream AUG-guided cryptic translational initiation modulation on the whole proteome dynamics in human cancer cells.

## Results

**Fig. 1:**
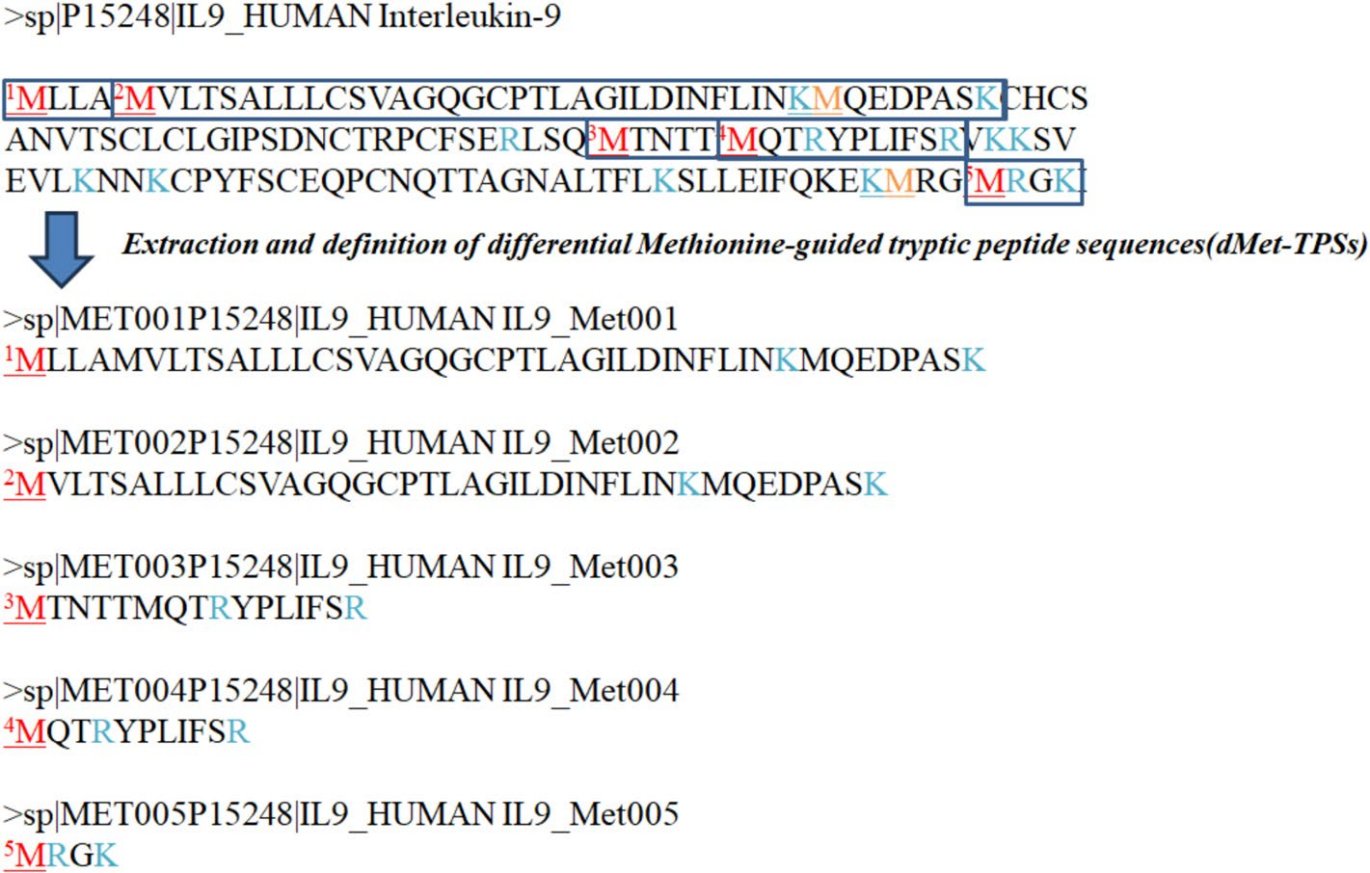
Construction of differential Methionine-guided Tryptic Peptide Sequence (dMet-TPS) Database for systematic identification of cryptic translation initiation sites. The computationally predicted N-terminal tryptic peptide fragments defined by differential AUG-guided translation initiation were extracted from each full-length amino acid sequence stored in the well-curated Swiss-Prot human protein database, with one missed tryptic cleavage allowed. The newly generated differential peptide sequence data on each protein entry were assigned with the unique accession defined by the methionine position of the alternative translation start site followed by the corresponding accession of the original Swiss-Prot protein entry. The representative definition of dMet-TPSs predicted from the Interleukin-9 (IL9) protein sequence (Swiss-Prot ID: P15248) was depicted with their corresponding amino acid sequences, respectively

In order to systematically identify alternative AUG-guided cryptic translation initiation sites on each protein coding region in our proteomic workflow, we newly generated and compiled peptide sequence data on alternative downstream AUG-guided tryptic peptide fragments as described in Fig. 1. The computationally predicted N-terminal tryptic peptide fragments defined by differential AUG-guided translation initiation were extracted from each full-length amino acid sequence stored in the well-curated Swiss-Prot human protein database [23], with one missed tryptic cleavage allowed (Fig. 1). The newly generated differential peptide sequence data on each protein entry were assigned with the unique accession defined by the methionine position of the translation start site followed by the original accession of the Swiss-Prot protein entry (Fig. 1, Additional file 1: Table S1). The above predicted N-terminal peptide sequence dataset was combined with the Swiss-Prot protein sequence database and applied for RTS-assisted comprehensive protein identification.

### Identification and quantification of EGF-regulated human proteins defined in the Swiss-Prot protein sequence database

**Fig. 2:**
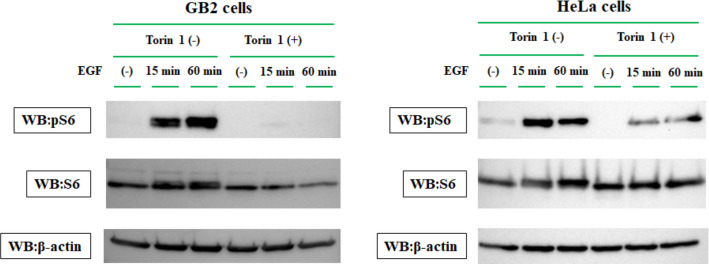
Quantitative evaluation of Torin 1-dependent translational perturbation of human cancer cells in response to EGF treatment. The human GB2 and HeLa cells were pretreated in the presence/absence of 250 nM Torin 1 for 120 min, serum-starved and treated with 20 ng/ml EGF for 15 min or 60 min, respectively. Western blots of phosphorylated RPS6 were applied for quantitative evaluation of Torin1-dependent translational perturbation in response to EGF stimulation. Beta-Actin was used as a loading control

**Fig. 3:**
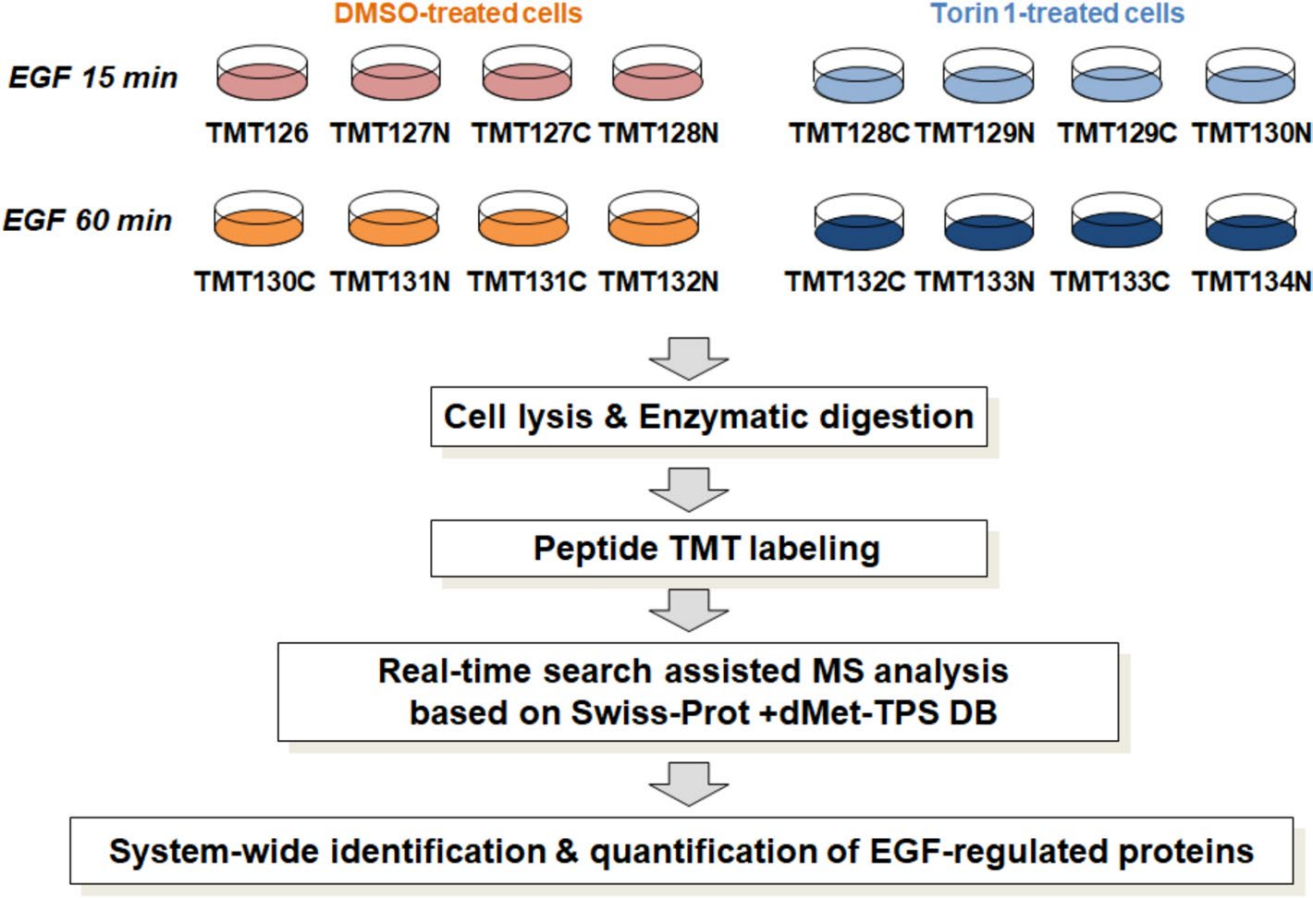
Schematic representation of RTS-dependent in-depth quantitative proteomic analysis of EGF-treated human cancer cells. The human GB2 and HeLa cells pretreated with DMSO or Torin 1 (250 nM) for 2 h were stimulated with EGF for 15 min/60 min. The cells were then lysed, tryptic digested and labeled with discrete TMT tags, respectively. The TMT-labeled peptide mixture was subjected to nanoLC-MS/MS analysis in an RTS-dependent data acquisition on Orbitrap Eclipse Tribrid mass spectrometer using the customized dMet-TPS database combined with the original Swiss-Prot human protein sequences. Protein identification and quantification were also conducted by searching against the above integrated protein sequence database

**Fig. 4:**
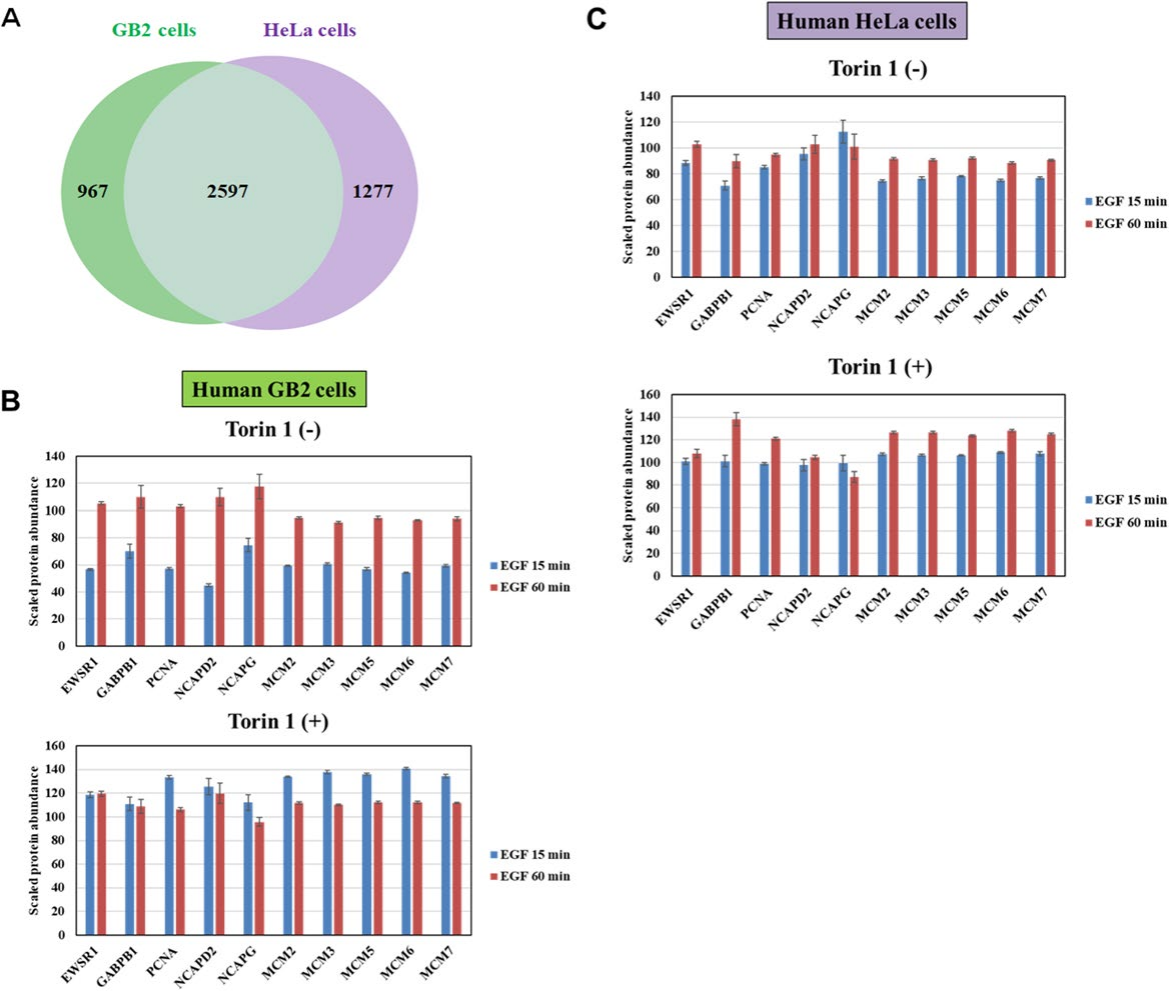
Identification and quantification of Swiss-Prot human protein sequences by RTS-assisted shotgun MS analysis. **A** Venn diagram of Swiss-Prot human protein sequences identified from human GB2 and HeLa cells in RTS-assisted in-depth proteomic analyses as described in Fig. 3. **B**, **C** Translational regulation of representative Swiss-Prot proteins in human GB2 and HeLa cells in response to EGF treatment. Four replicates with differential TMT signals were applied for quantitative evaluation of translational regulation in the presence/absence of Torin 1

EGF is well known to induce cell proliferation through EGF receptor-mediated signaling cascades, leading to ribosome-mediated activation of translation initiation in response to elevated phosphorylation of its subunit RPS6 through mTOR signaling activation [24]. In order to evaluate the effect of EGF stimulation on the phosphorylation status of RPS6, we performed an Western blot analysis of human GB2 and HeLa cells, which were previously characterized in the phosphoproteomic analysis of EGF signaling [10, 11]. Our result showed the phosphorylation level of RPS6 was dramatically increased in response to EGF administration in the absence of mTOR inhibitor Torin 1, while EGF-induced RPS6 activation was completely suppressed under Torin 1-based signaling perturbation in human GB2 cells (Fig. 2). For system-wide quantitative evaluation of the whole cellular proteome regulation triggered by EGF-mTOR signaling axis, we then performed a TMT-based large-scale proteomic analysis of EGF-induced human cancer cells using advanced RTS platform on Orbitrap Eclipse Tribrid MS system. The human GB2 and HeLa cells pretreated in the presence/absence of 250 nM Torin 1 for 120 min were stimulated with 20 ng/ml EGF for 15 min or 60 min, lysed, tryptic digested and labeled with discrete TMT tags as described in Fig. 3. Our RTS-assisted quantitative proteomic analysis of human cancer cells led to identification of more than 26,000 unique peptides derived from 4,841 Swiss-Prot human proteins in total (Fig. 4A). Among 3,564 protein sequences defined in Swiss-Prot human protein database, 114 protein molecules were found to be translationally upregulated in response to prolonged EGF treatment (from 15 to 60 min) with more than 1,fivefold change (*p* < 0.05) in human GB2 cells (Additional file 2: Table S2). Notably, the protein expression level of approximately 90% (101 out of 114) protein molecules, including transcription regulators (EWSR1, GABPB1) and cell cycle/cell division regulators (PCNA, Condensin complex subunits, MCM complex proteins), was quantitatively maintained in the presence of Torin 1, indicating that EGF-induced translational upregulation was systematically suppressed through mTOR signaling inhibition in human GB2 cells (Fig. 4B, Table 1). In contrast, the same quantitative evaluation of human HeLa cells revealed that only 9 protein molecules among 3,874 Swiss-Prot human proteins were translationally upregulated with more than 1,fivefold change (*p* < 0.05) in response to prolonged EGF treatment (Additional file 3: Table S3). Our analysis also revealed that 7 proteins out of these 9 regulators were upregulated even in the presence of Torin 1, indicating that a very limited fraction of the expressed proteome was translationally controlled via phosphorylated RPS6-dependent mechanisms in human HeLa cells (Table 1).

**Table 1:** List of Swiss-Prot defined proteins translationally upregulated in response to prolonged EGF treatment

Accession	Description	Fold Change (EGF 60 min/EGF 15 min)	Adjusted *p*-value
**Human GB2 Cells**			
Q9H1E3	Nuclear ubiquitous casein and cyclin-dependent kinase substrate 1	3.084	5.30048E-14
Q01105	Protein SET	2.973	5.30048E-14
Q15021	Condensin complex subunit 1	2.535	0.000036421
P39687	Acidic leucine-rich nuclear phosphoprotein 32 family member A	2.526	8.83842E-08
Q92688	Acidic leucine-rich nuclear phosphoprotein 32 family member B	2.397	5.30048E-14
Q9P0M6	Core histone macro-H2A.2	2.363	5.30048E-14
P68431	Histone H3.1	2.314	0.00542286
Q86WB0	Zinc finger C3HC-type protein 1	2.157	0.014663235
Q9HAV4	Exportin-5	2.094	1.05209E-05
Q9Y3E1	Hepatoma-derived growth factor-related protein 3	2.049	0.044042362
P37837	Transaldolase	2.02	4.4706E-09
Q01130	Serine/arginine-rich splicing factor 2	1.963	5.64653E-08
P29401	Transketolase	1.96	5.30048E-14
Q02543	Large ribosomal subunit protein eL20	1.93	0.000019191
Q9BTT0	Acidic leucine-rich nuclear phosphoprotein 32 family member E	1.93	0.046210641
P06400	Retinoblastoma-associated protein	1.911	1.03074E-06
Q9Y251	Heparanase	1.876	0.00035584
Q8WY36	HMG box transcription factor BBX	1.863	0.020584908
Q8WW12	PEST proteolytic signal-containing nuclear protein	1.86	5.30048E-14
Q6IA69	Glutamine-dependent NAD(+) synthetase	1.847	8.98585E-05
Q9Y5U9	Immediate early response 3-interacting protein 1	1.845	0.002694633
Q01844	RNA-binding protein EWS	1.843	5.30048E-14
Q9UNL2	Translocon-associated protein subunit gamma	1.837	0.00064327
P61956	Small ubiquitin-related modifier 2	1.833	0.001705907
Q6IN85	Serine/threonine-protein phosphatase 4 regulatory subunit 3A	1.82	1.34204E-08
Q9HA82	Ceramide synthase 4	1.819	0.015364485
P35659	Protein DEK	1.815	5.30048E-14
Q6UX53	Thiol S-methyltransferase TMT1B	1.785	0.003276857
Q9BV23	Monoacylglycerol lipase ABHD6	1.777	0.001931387
Q16656	Nuclear respiratory factor 1	1.775	0.009745977
P12004	Proliferating cell nuclear antigen	1.773	0.025112549
P02649	Apolipoprotein E	1.752	0.047673241
Q8NHH9	Atlastin-2	1.75	0.004388952
O94856	Neurofascin	1.744	0.000295305
Q92538	Golgi-specific brefeldin A-resistance guanine nucleotide exchange factor 1	1.731	0.026786244
Q9Y2S2	Lambda-crystallin homolog	1.72	1.09159E-09
Q14566	DNA replication licensing factor MCM6	1.713	3.58424E-11
Q9UBE0	SUMO-activating enzyme subunit 1	1.709	0.006236062
P14324	Farnesyl pyrophosphate synthase	1.7	3.98231E-05
O14980	Exportin-1	1.698	4.13119E-10
P51858	Hepatoma-derived growth factor	1.696	5.30048E-14
P55060	Exportin-2	1.685	0.001217187
Q9UPN6	SR-related and CTD-associated factor 8	1.684	0.000175895
Q9Y6D9	Mitotic spindle assembly checkpoint protein MAD1	1.679	3.92327E-05
P16949	Stathmin	1.674	5.30048E-14
Q13564	NEDD8-activating enzyme E1 regulatory subunit	1.662	3.47804E-06
Q8N0U8	Vitamin K epoxide reductase complex subunit 1-like protein 1	1.659	6.84149E-05
P49321	Nuclear autoantigenic sperm protein	1.651	0.000818416
Q8TBE1	Protein cornichon homolog 3	1.647	0.000153215
Q9NZL9	Methionine adenosyltransferase 2 subunit beta	1.642	0.033329996
O43670	BUB3-interacting and GLEBS motif-containing protein ZNF207	1.641	5.30048E-14
Q9NY33	Dipeptidyl peptidase 3	1.638	3.20395E-06
P33992	DNA replication licensing factor MCM5	1.631	0.044224359
O75367-1	Isoform 2 of Core histone macro-H2A.1	1.628	5.30048E-14
P53999	Activated RNA polymerase II transcriptional coactivator p15	1.626	2.78625E-10
P62861	Ubiquitin-like FUBI-ribosomal protein eS30 fusion protein	1.625	0.010296193
Q00796	Sorbitol dehydrogenase	1.623	0.000470052
P05455	Lupus La protein	1.621	1.81765E-05
Q9BPX3	Condensin complex subunit 3	1.618	0.017798488
P11766	Alcohol dehydrogenase class-3	1.613	4.62819E-08
P51688	N-sulphoglucosamine sulphohydrolase	1.613	9.09197E-05
Q9NSI6	Bromodomain and WD repeat-containing protein 1	1.61	3.58383E-05
Q96AE4	Far upstream element-binding protein 1	1.608	5.30048E-14
Q03519	Antigen peptide transporter 2	1.605	0.023573818
Q7Z5K2	Wings apart-like protein homolog	1.603	1.8692E-06
Q8N9R8	Protein SCAI	1.601	0.015617091
P31153	S-adenosylmethionine synthase isoform type-2	1.597	0.015968737
Q13442	28 kDa heat- and acid-stable phosphoprotein	1.594	0.007219881
Q92945	Far upstream element-binding protein 2	1.591	1.09601E-11
O00712	Nuclear factor 1 B-type	1.59	0.003592945
P09104	Gamma-enolase	1.588	0.009184064
P49736	DNA replication licensing factor MCM2	1.585	5.30048E-14
Q8WXF1	Paraspeckle component 1	1.585	0.007397103
P52209	6-phosphogluconate dehydrogenase, decarboxylating	1.584	5.30048E-14
P40429	Large ribosomal subunit protein uL13	1.58	5.30048E-14
Q29RF7	Sister chromatid cohesion protein PDS5 homolog A	1.576	5.30048E-14
Q6PCB5	Lysine-specific demethylase RSBN1L	1.576	0.002081715
P56937	3-keto-steroid reductase/17-beta-hydroxysteroid dehydrogenase 7	1.574	0.015823064
P26640	Valine–tRNA ligase	1.573	4.9982E-10
P33993	DNA replication licensing factor MCM7	1.568	1.8769E-10
P10768	S-formylglutathione hydrolase	1.567	0.028936872
Q14139	Ubiquitin conjugation factor E4 A	1.564	0.02246688
Q7Z3B3	KAT8 regulatory NSL complex subunit 1	1.553	5.71547E-05
Q9H553	Alpha-1,3/1,6-mannosyltransferase ALG2	1.55	0.00058787
Q06330	Recombining binding protein suppressor of hairless	1.543	1.90594E-11
Q13535	Serine/threonine-protein kinase ATR	1.539	0.000872688
Q9BZZ5	Apoptosis inhibitor 5	1.537	1.07993E-11
P63244	Small ribosomal subunit protein RACK1	1.536	8.01954E-06
Q06547	GA-binding protein subunit beta-1	1.534	0.022389343
Q9H8H3	Thiol S-methyltransferase TMT1A	1.527	5.30048E-14
Q9Y5L0-1	Isoform 1 of Transportin-3	1.525	0.023095943
Q9BXT8	RING finger protein 17	1.522	0.047682526
O95232	Luc7-like protein 3	1.519	5.80967E-11
P40938	Replication factor C subunit 3	1.518	0.001185046
P25205	DNA replication licensing factor MCM3	1.517	5.30048E-14
Q15436	Protein transport protein Sec23A	1.517	0.00053507
Q99986	Serine/threonine-protein kinase VRK1	1.514	0.001161646
P55786	Puromycin-sensitive aminopeptidase	1.513	0.000639273
P46776	Large ribosomal subunit protein uL15	1.511	0.000251103
O75925	E3 SUMO-protein ligase PIAS1	1.508	7.52E-04
Q8WX92	Negative elongation factor B	1.503	5.88068E-07
**Human HeLa Cells**			
Q8WY36	HMG box transcription factor BBX	1.7	0.027904483
Q9NP58	ATP-binding cassette sub-family B member 6	1.614	0.000866332

The proteins which showed FC > 1.5 specifically in the absence of Torin 1 were listed (Adjusted *p*-value < 0.05)

### Proteome-wide exploration for alternative AUG-guided novel proteoforms differentially translated in human cancer cells

In order to identify cryptic translation initiation sites defined by alternative AUG start codons on a proteome-wide scale, we newly generated an original amino acid sequence database which contained differential AUG-guided tryptic peptide fragments computationally predicted from Swiss-Prot human protein reference sequences as described in Fig. 1 and applied this customized database for RTS-assisted focused protein identification. In order to statistically evaluate the false discovery rate for novel peptide sequences as well as Swiss-Prot protein sequences, we combined these two sequence databases for Sequest-based peptide identification.

**Fig. 5:**
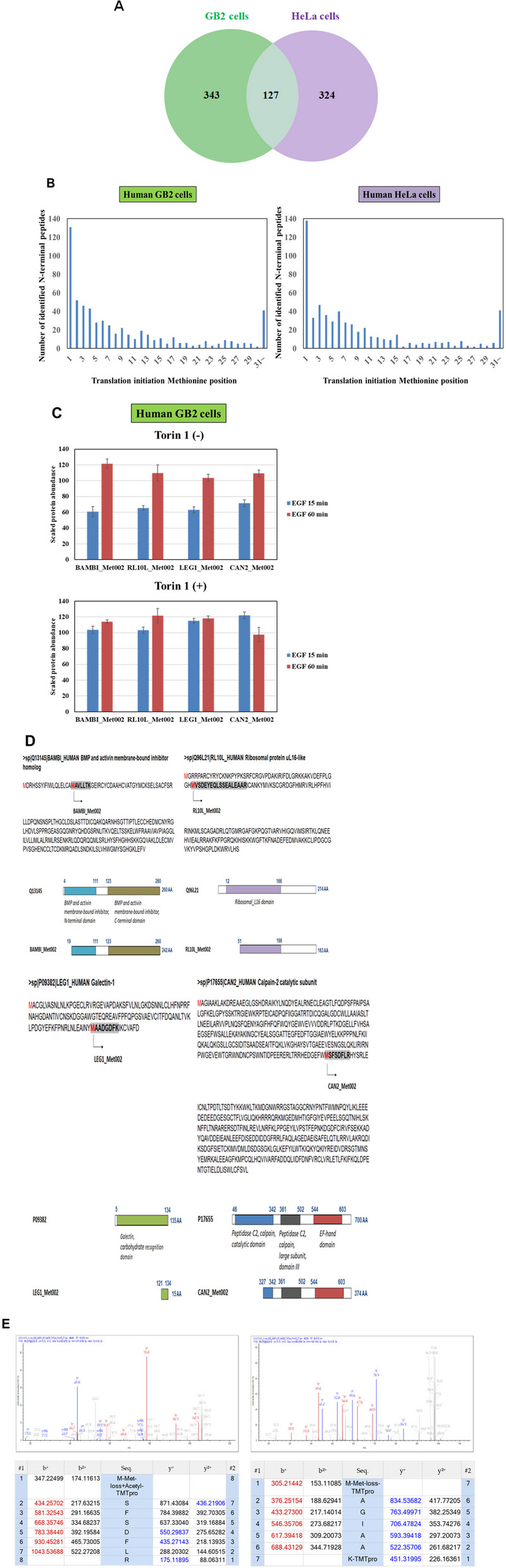
Identification and quantification of differential AUG-guided novel proteins by RTS-assisted shotgun MS analysis. **A** Venn diagram of differential AUG-guided novel proteins identified from human GB2 and HeLa cells in RTS-assisted in-depth proteomic analyses as described in Fig. 3. **B** Numerical distribution of identified N-terminal peptides translated from each differential methionine site. **C** Translational regulation of the second methionine-guided novel N-terminal proteins significantly regulated in human GB2 cells. Four replicates with differential TMT signals were applied for quantitative evaluation of DMSO/Torin 1 pretreated human GB2 cells in response to prolonged EGF treatment. **D** Primary domain structure of the second methionine-guided novel N-terminal proteins significantly regulated in human GB2 cells. The novel peptide sequences identified from the dMet-TPS database are highlighted as gray boxes, whereas the first and second methionine are indicated as red on each original Swiss-Prot amino acid sequence, respectively. The functional domains defined by InterPro [25] are also represented for each original Swiss-Prot protein sequence. **E** Representative mass spectra of the N-terminal peptides translated from the first methionine (left) and the second methionine (right) of the Calpain-2 catalytic subunit (CAN2) protein sequence (Swiss-Prot ID: P17655). M-Met-loss + Acetyl-TMTpro: TMTpro-labeled acetyl group after removal of N-terminal methionine; M-Met-loss-TMTpro: TMTpro after removal of N-terminal methionine

In addition to more than 26,000 unique peptides derived from the original Swiss-Prot human reference protein sequences, our RTS-based in-depth proteomic detection unveiled EGF-induced quantitative dynamics of 794 novel peptide fragments from human GB2 cells and HeLa cells in total (Fig. 5A). Regarding human GB2 cells, N-terminal acetylation frequency for canonical peptides was 16% (21 out of 132 unique peptides), whereas 7% (34 out of 487 unique peptides) of non-canonical peptides was detected as N-terminal acetylated forms. With respect to human HeLa cells, the frequency for canonical and non-canonical peptides was 16% (21 out of 131 unique peptides) and 9% (42 out of 462 unique peptides), which was comparable with those of human GB2 cells. These novel N-terminal peptide fragments were derived not only from the second or third methionine-initiated tryptic peptide sequences but also from the far downstream AUG-guided translated peptide fragments defined by diversed gene locus on the human genome (Fig. 5B, Additional file 2: Table S2). The extensive analysis of N-terminal heterogeneity also revealed that there were seven proteins (CAN2, DCC, DYHC1, GNS, HNRH1, KLHL9, WDR3) translated from different methionine sites in human GB2 cells, while as many as 15 proteins (ACSL3, AP2M1, CAN2, COPB, DCC, EXOC6, HNRPU, MYH16, NU160, PRKDC, RYR2, RYR3, SL9C1, SSRP1, UN13C) were found to be translated from different methionine positions in human HeLa cells (Fig. 5C). Among 470 novel N-terminal proteoforms identified from human GB2 cells, 12 protein molecules showed EGF-regulated upregulation with more than 1, fivefold change (*p* < 0.05) (Table 2). From human HeLa cells, we have also detected as many as 451 novel N-terminal proteoforms and only 2 protein molecules were found to be upregulated in response to EGF stimulation (Table 2, Additional file 3: Table S3). Our RTS-assisted multiplexed quantitation of these two human cancer cells revealed cell type-specific dynamics of EGF-induced cryptic translation initiation on a variety of canonical proteins, which might lead to widespread generation of disease-related immature protein products.

**Table 2:** List of differential downstream AUG-guided novel proteins translationally upregulated in response to prolonged EGF treatment

Accession	Description	Fold Change (EGF 60 min/EGF 15 min)	Adjusted *p*-value
**Human GB2 cells**			
MET004P09104	ENOG_Met004	2.618	3.17454E-06
MET006P06733	ENOA_Met006	2.104	0.000129558
MET002Q13145	BAMBI_Met002	2.099	0.000567638
MET004P07099	HYEP_Met004	1.852	0.000620402
MET004P15586-2	GNS_Met004	1.797	0.002005861
MET009Q00839-2	HNRPU_Met009	1.783	0.000477519
MET005Q96AE4	FUBP1_Met005	1.739	3.65E-07
MET003Q96B97-2	SH3K1_Met003	1.683	0.002673434
MET002Q96L21	RL10L_Met002	1.616	0.012435357
MET002P09382	LEG1_Met002	1.608	0.000299983
MET002P17655	CAN2_Met002	1.54	0.020183381
MET007P19532	TFE3_Met007	1.508	0.020323647
**Human HeLa cells**			
MET004P26641-2	EF1G_Met004	2.206	0.003365755
MET008Q13011	ECH1_Met008	1.598	0.004939439

The proteins which showed FC > 1.5 specifically in the absence of Torin 1 were listed (Adjusted *p*-value < 0.05)

## Discussion

RTS data acquisition platform enabled us to perform a robust identification of differential AUG-guided novel peptides through efficient selection of target precursor ions labeled with multiplexed isobaric reagents. As a result of high-throughput proteomic detection based on advanced RTS platform, our TMT-based multiplexed quantification of the whole proteome changes in response to prolonged EGF treatment unveiled system-wide translational upregulation of differential AUG-guided cryptic translation initiation of novel N-terminal proteoforms in addition to glioblastoma-related transcription factors (EWSR1, GABPB1) and a wide range of cell cycle/cell division regulators (PCNA, NCAPD2, NCAPG, MCM2, MCM3, MCM5, MCM6, MCM7) in human GB2 cells (Fig. 4B, Fig. 5D). The previous phosphoproteomic analysis of human GB2 cells clearly showed that the phosphorylation status of RPS6 was prominently upregulated after EGF treatment for 15 min, which might possibly reflect a cell-type specific translational regulation of the above proteins [11]. As shown in Fig. 4B, the above cell cycle/cell division regulators were prominently increased by EGF stimulation in human GB2 cells, but not in human HeLa cells, which indicated that EGF-induced translational regulation was quantitatively controlled in a cell-type specific manner. Very interestingly, among translationally regulated proteins which showed FC > 2 in response to prolonged EGF treatment (from 15 to 60 min), Nuclear ubiquitous casein and cyclin-dependent kinase substrate 1 (NUCKS) and Zinc finger C3HC-type protein 1 (ZC3C1) were also reported to be regulated upon EGF stimulation (15 min) at the phosphorylation level [11].

The previous report based on ribosome footprinting indicated that mTOR-dependent phosphorylation of RPS6 contributed to preferential translation of short coding sequences (CDSs) rather than longer CDSs [26]. Considering that alternative translation initiation from downstream AUG start codons could cause substantial deletion of functional domains on a variety of canonical proteins (Fig. 5E), our result suggested that mTOR-dependent phosphorylation of RPS6 in response to prolonged EGF treatment might also lead to widespread generation of immature protein products in human cancer cells. Regarding Gamma-enolase (ENOG), we extensively analyzed　EGF-induced interaction dynamics of Gamma-enolase protein complexes through immunoprecipitation using anti-Gamma-enolase monoclonal antibodies designed to immunoreact with the C-terminus of human Gamma-enolase protein. Shotgun proteomic analysis of affinity-purified Gamma-enolase protein complexes revealed that the first AUG-guided corresponding protein was decreased in response to prolonged EGF treatment (from 15 to 60 min), whereas downstream AUG-guided Gamma-enolase (ENOG_Met004) was quantitatively increased in human GB2 cells (Table 3). With respect to Alpha-enolase (ENOA), which is known to mutually interact with Gamma-enolase [27], two differential AUG-guided proteoforms (Alpha-enolase and ENOA_Met006) were found to react with Gamma-enolase and quantitatively maintained after prolonged EGF stimulation. This result suggested that EGF-induced translational regulation of differential AUG-guided protein products might contribute to heterogeneity of Gamma-enolase protein complex components in human GB2 cells. Further functional analysis of these novel N-terminal truncated proteins will be needed to clarify the systematic impact of EGF-induced translational modulation on cancer cell fate regulation.

**Table 3:** LFQ-based quantitative proteomic analysis of Gamma-Enolase protein complex components in response to prolonged EGF treatment

Protein description	Identified peptide sequence	Amino acid residue position^a^	Fold Change (EGF 60 min/EGF 15 min)
Gamma-Enolase	GNPTVEVDLYTAK	P09104 [16–28]	0.619
ENOG_Met004	MILPVGAESFR	P09104 [169–179]	2.096
Alpha-Enolase	GNPTVEVDLFTSK	P06733 [16–28]	1.07
ENOA_Met006	MILPVGAANFR	P06733 [169–179]	1.14

^a^The amino acid residue position on the corresponding Swiss-Prot protein sequence is represented along with the Swiss-Prot accession

## Conclusions

This study demonstrated that the ultra-deep mass spectrometric analysis based on the RTS platform on Orbitrap Eclipse Tribrid mass spectrometry system led to identification of approximately 27,000 peptides including non-canonical peptide fragments defined by alternative downstream translation initiation as well as already annotated human protein coding sequences and also allowed us to comparatively evaluate EGF-dependent proteome regulation in human GB2 and HeLa cells. Our methodology provided the first proteome-wide evidence of cryptic translational initiation modulation in human cancer cells and thus will also be widely applicable for evaluating drug-perturbed translation initiation dynamics in every biological context.

## Methods

### Reagents and antibodies

Epidermal growth factor (EGF), and basic fibroblast growth factor (bFGF) were obtained from Wako (Osaka, Japan). Torin 1 was purchased from Cayman Chemical (Ann Arbor, MI) and used as dimethylsulfoxide (DMSO) solution. Anti-Phospho-S6 Ribosomal Protein (Ser235/236) (D57.2.2E) XP® Rabbit mAb (4858), Anti-S6 Ribosomal Protein (5G10) Rabbit mAb (2217), Anti-β-Actin (13E5) Rabbit mAb (4970), Anti-Enolase-2 (D20H2) Rabbit mAb (8171) and Anti-rabbit IgG HRP-linked Antibody (7074) were obtained from Cell Signaling Technology (Danvers, MA). Protein A Magnetic Beads (73,778) and 10 × Cell Lysis Buffer (9803) were also purchased from Cell Signaling Technology (Danvers, MA). Synthetic peptides for AGIAAK and SFSDFLR with N-terminal acetylation were obtained from Cosmo Bio, Tokyo, Japan (Additional file 4: Fig S1).

### Cell culture and drug perturbation

Glioblastoma initiating cells were originally established from the glioblastoma brain tissues in the University of Tokyo Hospital based on the written informed consent to undertake genetic and molecular analyses from the patients, which was approved by the Research Ethics Committee at the Institute of Medical Science, The University of Tokyo (2024–56–1002). The glioblastoma patient-derived GB2 cells, which had been already established and analyzed in a series of our previous studies [11, 28, 29], were cultured in Dulbecco’s modified Eagle’s medium: Nutrient Mixture F-12 (DMEM/F12) media (Pierce) with 2% B27 supplement minus vitamin A, 20 ng/ml EGF and 20 ng/ml basic fibroblast growth factor (bFGF) as previously described [11]. Human cervical cancer HeLa cells were cultured in Dulbecco’s modified Eagle’s medium (DMEM) media containing 10% fetal bovine serum (FBS). These cancer cells were pretreated in the presence/absence of 250 nM Torin 1 for 120 min, serum-starved and then treated with 20 ng/ml EGF for 15 min or 60 min. The cells were then washed three times with PBS, harvested, and suspended in 8 M urea containing Benzonase (Novagen, Madison, WI).

### TMT Labeling

The cell lysates were quantified using BCA Protein Assay Kit (Thermo Fisher Scientific, Waltham, MA) and 10 µg of each cell lysate was labeled with TMTpro^TM^16plex (Thermo Fisher Scientific, Waltham, MA) according to the manufacturer’s instruction. Briefly, the proteins were reduced with 10 mM tris (2-carboxyethyl) phosphine (TCEP) for 60 min at 56 °C, alkylated with 17 mM iodoacetamide for 30 min. After precipitation with methanol/chloroform, the proteins were reconstituted in 100 mM triethylammonium bicarbonate (TEAB) and digested with Lysyl Endopeptidase, Mass Spectrometry Grade (FUJIFILM Wako Chemicals, Osaka, Japan) for 5 h and then with Trypsin Gold, Mass Spectrometry Grade (Promega, Madison, WI) overnight at 37 °C. The fragmented peptide mixtures were then labeled with distinct TMT reagents, mixed and desalted by ZipTip C18 (Millipore, Billerica, MA).

### Database construction

The computationally predicted N-terminal tryptic peptide fragments defined by differential AUG-guided translation initiation were extracted from each full-length amino acid sequence stored in the well-curated Swiss-Prot human protein database, with one missed tryptic cleavage allowed (Fig. 1). The newly generated differential peptide sequence data on each protein entry were assigned with the unique accession defined by the methionine position of the translation start site followed by the original accession of the Swiss-Prot protein entry (Fig. 1, Additional file 1: Table S1).

### LC–MS/MS analysis

Shotgun proteomic analyses were performed by Orbitrap Eclipse Tribrid mass spectrometer with FAIMS Pro interface (Thermo Fisher Scientific, Waltham, MA), which was connected to Vanquish Neo UHPLC system (Thermo Fisher Scientific, Waltham, MA). The peptide samples were separated using a linear gradient of 2–24% mobile phase (0.1% formic acid in acetonitrile) at 300 nl/min. Full-scan MS spectra were acquired with a resolution of 120,000 and subsequent MS/MS scans were performed in the ion trap using collision-induced dissociation (CID) fragmentation with a normalized collision energy of 35% with 10 ms maximum injection time. Swiss-Prot human protein sequences were extracted from UniProt human reference proteome (UP000005640) and combined with their alternative AUG-guided non-canonical predicted peptide sequences (Additional file 1: Table S1) to perform an RTS-assisted data acquisition on Orbitrap Eclipse Tribrid mass spectrometer. RTS data acquisition platform has been implemented with Orbitrap Eclipse Tribrid mass spectrometer and enabled robust and accurate multiplexed quantification in combination with synchronous precursor selection (SPS)-MS3 acquisition of isobaric tag-based reporter ions [30]. In the RTS-MS3 method, MS2 spectra were subjected to RTS using the settings as indicated below; Trypsin was set as enzyme (cleavage next to arginine or lysine, but not before proline). Static modifications were TMTpro16plex on Lysine (K) and N-Terminus in addition to carbamidomethylation on cysteine (C). Oxidation of methionine (M) was set as a variable modification. Maximum missed cleavages were set to 1 and maximum variable modifications to 2. The measurements were repeated twice and merged in the subsequent data analysis using Proteome Discoverer Software (version 3.1) (Thermo Fisher Scientific, Waltham, MA).

### Protein identification and quantification

Protein identification was conducted by searching against the above specialized database of Swiss-Prot human protein sequences in combination with their alternative AUG-guided non-canonical predicted peptide sequences (Additional file 1: Table S1) using Sequest HT algorithm in Proteome Discoverer Software (version 3.1) (Thermo Fisher Scientific, Waltham, MA). Cysteine carbamidomethylation and TMTpro on Lysine (K) and N-Terminus were set as fixed modifications, whereas methionine oxidation and protein N-terminal acetylation were set as variable modifications. A maximum of two missed cleavages was allowed in our database search, while the mass tolerance was set to 10 ppm for peptide masses and 0.6 Da for MS/MS peaks, respectively. Regarding the RTS-assisted measurement using Orbitrap Eclipse Tribrid mass spectrometry system, MS2 data acquisition was conducted in the linear ion trap to achieve highly sensitive detection, whereas high-resolution scan in the orbitrap was applied during MS1 and MS3 scans to measure accurate precursor masses and resolve the reporter ions for quantification, respectively. Therefore, the parameters for mass tolerance were set according to the recent papers in which RTS-assisted multiplexed quantification was also conducted using the same mass spectrometry platform [13, 14]. In the process of peptide identification, we applied a filter to satisfy a false discovery rate < 1% at the PSM and peptide level. For TMT-based quantitative proteomic analysis of EGF-induced translational regulation, four replicates regarding DMSO/Torin 1 treated human cancer cells were measured to calculate log2-transformed fold change and log10-transformed p-value, which was adjusted via the Benjamini–Hochberg method. The four independent quantitative data on differential TMT signals was averaged and evaluated as the fold change of each protein amount in response to prolonged EGF stimulation (from 15 to 60 min).

### Western blotting analysis

Human GB2 and HeLa cells were cultured and drug-perturbed in the same manner as the sample preparation for mass spectrometric analysis. After the cells were lysed in the lysis buffer (8 M Urea, 500 mM Tris–HCl, pH 8.2), the cell lysates were quantified using BCA Protein Assay Kit (Thermo Fisher Scientific, Waltham, MA) and equal amounts of the cell lysates were separated on SDS-PAGE and transferred to a PVDF membrane. The membrane was probed with each primary antibody and then with the corresponding HRP-conjugated secondary antibody according to the protocol recommended by the manufacturer of each antibody. The blots were exposed to　Luminata™ Forte Western HRP Substrate (Merck KGaA, Darmstadt, Germany) and analyzed by iBright FL1500 imaging system (Thermo Fisher Scientific, Waltham, MA) as preciously described [15].

### Immunoprecipitation-mass spectrometry (IP-MS) analysis

Human GB2 cells were serum-starved and treated with 20 ng/ml EGF for 15 min or 60 min. After the cell lysates were incubated overnight at 4 °C with Anti- Enolase-2 (D20H2) Rabbit mAb, which was designed to immunoreact with the C-terminus of human Enolase-2, also known as Gamma-enolase, Protein A Magnetic Beads were added to purify protein complex components interacted with Gamma-enolase. The beads were then washed five times with 1 × Cell Lysis Buffer (Cell Signaling Technology), rinsed twice briefly with 50 mM Tris–HCl (pH 8.2) and subjected to on-beads digestion using Trypsin Gold, Mass Spectrometry Grade (Promega, Madison, WI) overnight at 37 °C. The fragmented peptide mixtures were desalted by ZipTip C18 (Millipore, Billerica, MA) and subjected to shotgun proteomic analyses using Vanquish Neo UHPLC system (Thermo Fisher Scientific, Waltham, MA) coupled with Orbitrap Eclipse Tribrid mass spectrometer with FAIMS Pro interface (Thermo Fisher Scientific, Waltham, MA) as described above. Peptides were separated on a reversed-phase column using a linear gradient of 2–24% acetonitrile in 0.1% formic acid at a flow rate of 300 nL/min. Full MS scans were acquired in the Orbitrap at a resolution of 120,000, followed by MS/MS scans in the ion trap using higher-energy collisional dissociation (HCD) with a normalized collision energy of 35% and a maximum injection time of 10 ms. Protein identification was conducted using the same parameter setting except for TMT labels and Label-Free Quantification (LFQ) was then performed using the Minora Feature Detector node in Proteome Discoverer Software (version 3.1) (Thermo Fisher Scientific, Waltham, MA) to evaluate the comparative amount of each protein molecule in response to differential EGF stimulation.

## Supplementary Information

Additional file 1: Table S1. List of differential AUG-guided tryptic peptide sequences extracted from Swiss-Prot human protein sequences.

Additional file 2: Table S2. TMT-based multiplex quantification of the whole proteome changes in DMSO/Torin 1-treated human GB2 cells in response to EGF treatment.

Additional file 3: Table S3. TMT-based multiplex quantification of the whole proteome changes in DMSO/Torin 1-treated human HeLa cells in response to EGF treatment.

Additional file 4: Fig S1. Representative mass spectra of the synthetic peptideschemically modified with TMTpro.

## Data Availability

The mass spectrometry proteomics data have been deposited to the ProteomeXchange Consortium via the jPOST repository with the dataset identifiers (PXD064188 and PXD070141) [31, 32].
