# Supplementary material for: Real-time search-assisted multiplexed quantitative proteomics reveals system-wide cryptic translation initiation in human cancer cells
Source: Genome Biol. 2026 May 28;27:240. doi: 10.1186/s13059-026-04120-z (PMC13404309; doi:10.1186/s13059-026-04120-z)
Supplement: Supplementary file 4 — Additional file 4: Fig S1. Representative mass spectra of the synthetic peptideschemically modified with TMTpro. [file 13059_2026_4120_MOESM4_ESM.pdf]

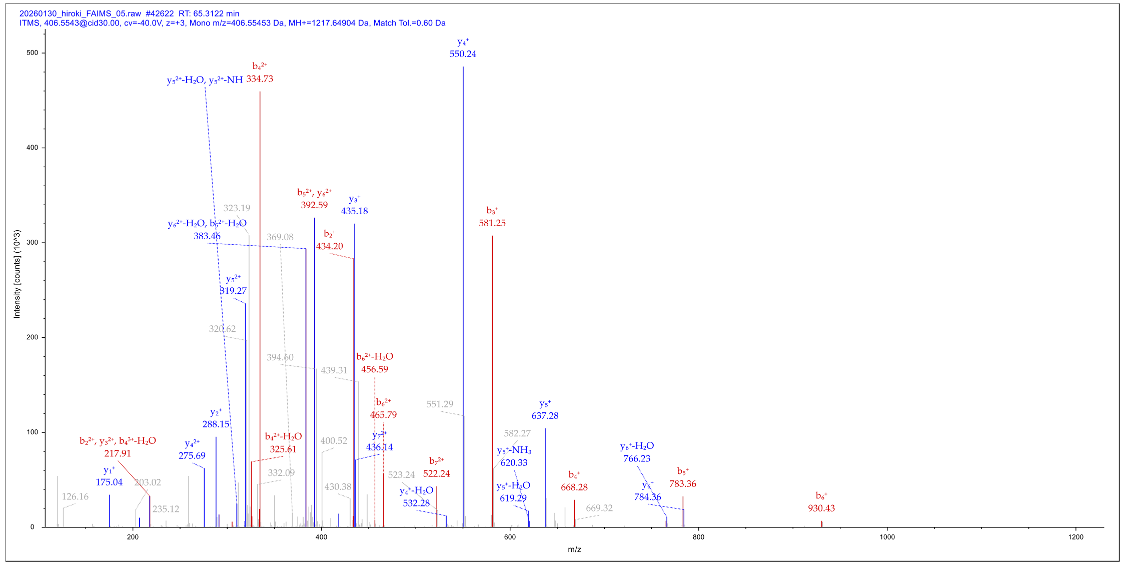

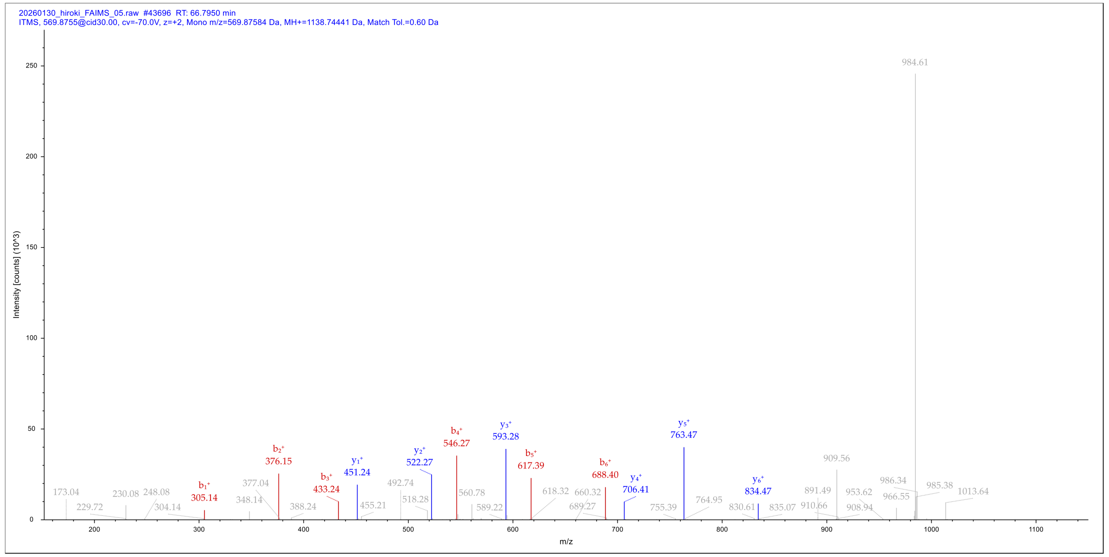

#1	b⁺	b²⁺	b³⁺	Seq.	y⁺	y²⁺	y³⁺	#2
1	347.22499	174.11613	116.41318	M-Met-loss+Acetyl -TMTpro				8
2	434.25702	217.63215	145.42386	S	871.43084	436.21906	291.14847	7
3	581.32543	291.16635	194.44666	F	784.39882	392.70305	262.13779	6
4	668.35746	334.68237	223.45734	S	637.33040	319.16884	213.11498	5
5	783.38440	392.19584	261.79965	D	550.29837	275.65282	184.10431	4
6	930.45281	465.73005	310.82246	F	435.27143	218.13935	145.76199	3
7	1043.53688	522.27208	348.51714	L	288.20302	144.60515	96.73919	2
8				R	175.11895	88.06311	59.04450	1

#1	b⁺	b²⁺	Seq.	y⁺	y²⁺	#2
1	305.21442	153.11085	M-Met-loss-TMTpro			7
2	376.25154	188.62941	A	834.53682	417.77205	6
3	433.27300	217.14014	G	763.49971	382.25349	5
4	546.35706	273.68217	I	706.47824	353.74276	4
5	617.39418	309.20073	A	593.39418	297.20073	3
6	688.43129	344.71928	A	522.35706	261.68217	2
7			K-TMTpro	451.31995	226.16361	1

Fig S1. Representative mass spectra of the synthetic peptides (AGIAAK and SFSDFLR with N-terminal acetylation) chemically modified with TMTpro

50 ng of each peptide was subjected to TMT labeling reaction and analyzed by nanoLC-MS/MS. M-Met-loss+Acetyl-TMTpro: TMTpro-labeled acetyl group after removal of N-terminal methionine; M-Met-loss-TMTpro: TMTpro after removal of N-terminal methionine.
